# Italian guidance on Dementia Day Care Centres: A position paper

**DOI:** 10.1007/s40520-023-02356-4

**Published:** 2023-02-16

**Authors:** Enrico Mossello, Marco Baccini, Francesca Caramelli, Carlo Adriano Biagini, Alberto Cester, Luc Pieter De Vreese, Gianluca Darvo, Claudio Vampini, Mabel Gotti, Andrea Fabbo, Alessandra Marengoni, Maria Chiara Cavallini, Guido Gori, Rabih Chattat, Monica Marini, Davide Ceron, Alessandro Lanzoni, Paolo Pizziolo, Andrea Mati, Iole Zilli, Claudia Cantini, Veronica Caleri, Elisabetta Tonon, David Simoni, Patrizia Mecocci, Andrea Ungar, Giulio Masotti, the Italian Group on Dementia Day Care Centres

**Affiliations:** 1 Department of Experimental and Clinical Medicine, Research Unit of Medicine of Ageing, University of Florence, Florence, Italy; 2 IRCCS Don Gnocchi Foundation, Florence, Italy; 3 Division of Geriatric Medicine, Azienda USL Toscana Centro, Pistoia, Italy; 4 Geriatrician and Physiatrist, Padua, Italy; 5 Department of Mental Health and Addictions, Cognitive Clinic, Azienda USL Modena, Italy; 6 Department of Architecture, University of Florence, Florence, Italy; 7 Psychiatric and Psychogeriatric Service, San Francesco Hospital, Garofalo Health Care, Verona, Italy; 8 Psychotherapist and Psychoanalyst, Italian Society of Interpersonal Psychoanalysis, Florence, Italy; 9 Cognitive Disorders and Dementia Unit, Health Authority and Services of Modena, Modena, Italy; 10 Department of Clinical and Experimental Sciences, University of Brescia, Brescia, Italy; 11 Continuity of Care Agency, Department of Geriatrics, Azienda Ospedaliero-Universitaria Careggi, Florence, Italy; 12 Scientific Director, PAS Pubbliche Assistenze Foundation, Florence, Italy; 13 Department of Psychology, University of Bologna, Bologna, Italy; 14 Staff Coordinator, Healthcare Executive, Tuscany Region, Italy; 15 Opera Immacolata Concezione Foundation, Padua, Italy; 16 Cognitive Disorders and Dementia Unit, Primary Care Department, Health District of Modena, Modena, Italy; 17 Anglia Ruskin University, Cambridge, UK; 18 Piante Mati, Pistoia, Italy; 19 Cooperativa Elleuno, Firenze, Italy; 20 IRCCS Don Gnocchi Foundation, Florence, Italy; 21 Health Area Manager, Arnera Cooperativa Sociale, Pontedera, Italy; 22 Section of Gerontology and Geriatrics, Department of Medicine, University of Perugia, Perugia, Italy

**Keywords:** Dementia, Dementia day care centre, Long-term care, Behavioural symptoms, Caregiver

## Abstract

Dementia Day Care Centres (DDCCs) are defined as services providing care and rehabilitation to people with dementia associated with behavioural and psychological symptoms (BPSD) in a semi-residential setting. According to available evidence, DDCCs may decrease BPSD, depressive symptoms and caregiver burden. The present position paper reports a consensus of Italian experts of different disciplines regarding DDCCs and includes recommendations about architectural features, requirements of personnel, psychosocial interventions, management of psychoactive drug treatment, prevention and care of geriatric syndromes, and support to family caregivers. DDCCs architectural features should follow specific criteria and address specific needs of people with dementia, supporting independence, safety, and comfort. Staffing should be adequate in size and competence and should be able to implement psychosocial interventions, especially focused on BPSD. Individualized care plan should include prevention and treatment of geriatric syndromes, a targeted vaccination plan for infectious diseases including COVID-19, and adjustment of psychotropic drug treatment, all in cooperation with the general practitioner. Informal caregivers should be involved in the focus of intervention, with the aim of reducing assistance burden and promoting the adaptation to the ever-changing relationship with the patient.

## Introduction

The establishment of Dementia Day Care Centres (DDCCs) dates back to 1979 in the UK [1]. Across the years, several scientific data have supported the use of semi-residential care for people with dementia, despite some limitations such as heterogeneous care models, diverse outcome measures and lack of randomized intervention studies. The large majority of studies showed efficacy in reducing depressive symptoms [2, 3] and behavioural and psychological symptoms in dementia (BPSD) [2–6] as well as in stimulating preserved cognitive abilities [4], although a few less recent studies reported no significant effects [7, 8]. Several studies found that semi-residential care may also represent a valuable support of family caregivers, reducing caregiver burden [2, 3, 9–11], although a systematic review on this topic concluded that, taken as a whole, literature data are ambiguous [12].

Several factors may explain the conflicting data, including the number of days/hours the participants attended the DDCC (is some cases only 1–2 days per week). Some patient and caregiver-related factors can limit the frequency of utilization of this service, including perceived loss of autonomy, denial, guilt or economic constraints [13]. Context factors, such as poor availability, social system complexity or lack of adequate information may further decrease the access [13]. The quality and types of psychosocial interventions delivered (the less recent of the cited articles generally used 'low-tech' interventions) also could impact on the results, as well as methodological constraints. Indeed, with these patients, equivalence between the comparison groups is difficult to achieve, and randomization is also often impossible [14]. Moreover, in some studies participants of the control group received some amount of treatment. For example, control caregivers in the study of Zank and Schacke [4] received respite care at home, so they did not meaningfully differ from caregivers using adult day care for their relative.

Semi-residential care can decrease the risk of hospitalization [8], possibly through the prevention of BPSD exacerbations and the monitoring of chronic pathologies and geriatric syndromes. Whether it would also help to postpone the dementia patient's admission to a nursing home is still a matter of debate. Wimo et al. showed that attending a DDCC could avoid or delay permanent institutionalization [7], but other studies did not replicate or even reverse this finding [8, 15, 16]. However, admission to a nursing home may be independent of the patient's condition and the caregiver's stress and may be due to contingencies (e.g. caregiver illness) or to caregiver age (13). Other unmeasured factors such as an increased propensity to institutionalization when the patient starts spending a lot of time away from home might also play a role (15,16). In this respect, the effect might also depend on when the patient enters the DDCC, i.e. whether in the advanced stages of the disease, when the patient is severely impaired or at an earlier stage. In the first case, caregivers may be so burdened that the relief provided by DDCC might actually hasten institutionalization [17].

In Italy, the first experiences of semi-residential care for older subjects date back to the 1980s, with social services dedicated to older subjects with preserved functional autonomy, while the first day care centres for disabled older subjects affected by dementia date back to the 1990s, in the Lombardia, Emilia-Romagna and Toscana regions [18]. The presence of DDCCs has been subsequently included among “Essential levels of assistance” guaranteed by the Italian National Health Service for people with dementia in need of “rehabilitation, reorientation and safeguarding” [19]. With regard to funding, the Italian National Health Service covers the healthcare costs of the service (e.g. nursing, physiotherapy), which represents 50% of the total fee, while social costs (including basic daily care and accommodation) are charged to the person with dementia and his/her family, in proportion to the income, and the remaining share is covered by the municipality [20, 21].

In the present document, DDCCs are defined as *semi-residential structures aimed at providing social and health care for a specific target of people with dementia, namely those suffering from clinically significant BPSD who require assistance and rehabilitation approach for a definite period of time.* People who attend DDCCs should preferably be able to walk, independently or with assistance.

Since 2011, the “Italian Group on Dementia Day Care Centres”, a multidisciplinary group of professionals involved in geriatric medicine, has been working on drafting an expert consensus on day care for older subjects with dementia [22]. This article presents the recommendations developed by the experts’ panel.

The following chapters include a summary of the guidance regarding different areas of care analysed by the authors, namely care pathway, care environment, requirement of personnel, main psychosocial interventions, use of psychoactive treatment, management of geriatric syndromes, infection prevention, and support to family and caregivers.

## Methods

The guidance presented in this article has been developed by a multidisciplinary panel of experts including geriatricians, psychiatrists, psychologists, architects, nurses, physiotherapists, occupational therapists, social health educators and music therapists. All panellists were professionals with many years of experience in long-term care for dementia and/or experts in research methodology. Multiprofessional subgroups were set up with the aim of searching the literature and developing guidance on specific issues:

- DDCC definition, norms, and care pathway
- Physical environment
- Personnel
- Psychosocial interventions
- Drug treatment
- Geriatric syndromes
- Caregiver support

The experts were not asked to compulsorily conduct a systematic literature review with specific keywords, because the purpose of the work was not to develop a formal guideline. The proposals from each subgroup were discussed by the entire panel at subsequent meetings on the sidelines of the Italian conference on Alzheimer's day care centres. The work started in 2011 and since then the text has undergone numerous revisions and updates, which were also necessitated by advances in scientific research in this area.

The outbreak of the COVID-19 pandemic slowed the panel's work and also prompted the addition of specific recommendations on the prevention of infections. Following the COVID-19 pandemic, several DDCCs have also adopted instruments of telecare, including tele-monitoring with information–communication technology techniques and the use of technology to foster social inclusion and provide remote psychosocial and rehabilitative interventions. Although the authors acknowledge the importance of this promising field, they also believe that the available literature is not sufficient yet to provide a guidance on telecare in DDCCs.

## Care pathway for dementia day care recipients

The admission to DDCCs should follow an assessment of the patient by a memory clinic [23, 24] and a comprehensive assessment by the healthcare district commission about the appropriateness of admission, which usually decides for a limited (3–6 months), renewable period of attendance. Once the admission is confirmed, an individualized care plan (ICP) is drawn up by the DDCC staff, in agreement with family members and the general practitioner (GP). The ICP includes clinical, social, psycho-affective and functional problems identified during the comprehensive geriatric assessment; identifies the care needs and the strategies to pursue assistance targets; establishes regular checks, aimed at evaluating the results achieved and/or to update objectives or strategies [23, 24].

We recommend that the admission to DDCCs be gradual and preceded by a contact with family members. The ICP must guarantee social inclusion, psychosocial interventions, physical activity and nursing and rehabilitative interventions as needed, to be performed individually or in small groups. If necessary, the discharge from the service can be followed by a home care assistance aimed at facilitating the return at home or the possible transfer to a different long-term care service.

## Environment as a therapeutic intervention

DDCCs architectural features should be based on the peculiar needs of people with dementia with the purpose of providing adequate conditions of safety and comfort, supporting their independence, and creating a meaningful and stimulating environment. Scientific evidence supports the significant impact of environment on both health and well-being of people with dementia [25]. Particularly, an adequate living arrangement seems able to reduce the occurrence of behavioural disorders, to maintain preserved functional capacities and to support spatial and temporal orientation. Therefore, the environment could become an important pillar of assistance together with pharmacological and psychosocial treatments [26, 27]. Conversely, unsuitable environmental characteristics can trigger or increase behavioural disorders [28, 29]. An environmental element with a specific psychosocial purpose is represented by therapeutic garden that may help to decrease BPSD [30–32]. Moreover, the facility must be provided with an infirmary, in order to enable an adequate first aid in case of sudden injury or illness.

Building design must also take into account the needs of care team to facilitate their work and to reduce physical and emotional burden. The DDCCs should be fully accessible, possibly located within the city centre, next to community meeting places, to foster integration in social context.

Minimal structural requirements of semi-residential services for older subjects with disability include: localization in places easy to reach, to support social inclusion, availability of spaces devoted to socialization and common activities, availability of at least one accessible bathroom for every 10 care recipients and a maximum capacity of 30 care recipients [33]. In keeping with the minimal requirement of 40–45 sq m per resident in residential care services for disabled older adults [34] and subtracting the minimum surface of overnight spaces, we advise a minimum surface area of 25–30 sq m for each day care recipient.

Table 1 summarizes the main design features that should be respected for the realization of the DDCCs, in parallel with specific impairments and needs of people with dementia that should be addressed.

**Table 1:** Needs and/or impairments of people with dementia and relative recommendations of DDCCs design

Needs and/or impairments of people with dementia	Design recommendations
*Safety*	
Reduced awareness of environmental risks [35]	Eliminate physical and psychological barriers, both in indoor and outdoor spaces. Forbid or hide non-controlled areas or spaces that could represent a source of potential danger [35]
*Usability and space–time orientation*	
Inability to use space properly, resulting in anxiety, agitation and aberrant behaviours [36]	Eliminate physical and perceptual barriers that may limit the use of spaces and objects. Use implicit and explicit signage to ease orientation and identification of spaces and objects [36]
*Familiarity, comfort*	
Worsening social interaction associated with impersonal environment and furnishings [36–38]	Design spaces with domestic appearance and size [36–38]
*Environmental well-being, sensory aspects*	
Orientation, autonomy and mood affected by environmental characteristics (lighting, noise, temperature and odour) [28, 36, 39]	Provide suitable lighting levels for the type of activity carried out, allow for the regulation of temperature, the dispensation of aromas and the presence of environmental stimuli, such as music, images and colours. Reduce noise level [28, 36, 39]
*Flexibility*	
Time-dependent variations of needs of people with dementia and caregivers [40]	Flexibility and adaptability of spaces, according to the degree of disability, type of activities carried out and daily organization [40]
*Occupational well-being*	
Increasing work and psychological burden of care team due to environmental characteristics [40]	Provide spaces for the exclusive use of staff that allow visual control of care recipients’ spaces. The configuration of the space and the type of furnishings must facilitate care and therapeutic activities, indoor and outdoor [26, 40]
*Outdoor spaces (therapeutic garden)*	
Psychological and behavioural well-being associated with the contact of natural elements and physical activity [31–33]	Provide safe spaces, which facilitate physical activity, orientation and control by the staff. Seed native plants to facilitate recognition of the seasons and sensory stimulation (sight, smell, hearing, touch, aromas and fountain with running water) [31–33]
*Emergency and health care services*	
First aid for acute injuries and illnesses	An infirmary must be provided, with adequate equipment (examination bed; sphygmomanometer; stethoscope; thermometer; pulse oximeter; glucometer; bladder catheter kit; portable oxygen tanks; BLS-D kit; AED; EKG unit)

*DDCCs* dementia day care centres, *BLS-D*  basic life support defibrillation; AED automated external defibrillator, *EKG* electrocardiogram

## Personnel of DDCCs and their roles

The working group in DDCCs includes social and healthcare personnel, with specific skills in caring for older people with dementia and BPSD, capable of working together as a team. Aims of staff activity include taking care of day care recipients, promoting their interaction with each other, supporting caregivers, maintaining contacts with the socio-cultural entities outside DDCCs and cooperating with local services and GPs.

Minimal organization requirements of semi-residential services for disabled older subjects [33] include the presence of a service coordinator and the predisposition of an ICP for each care recipient. Specific qualifying standards for DDCC include a minimum of 80 min of total assistance per care recipient per day and the availability of nurse, nursing assistant and psychologist [19]. A more recently approved official document [41] includes a list of different professional roles involved in dementia care, whose presence we recommend in a DDCC (Table 2).

**Table 2:** Professional roles and activities within the care team

Professional role	Expected workload (hours per week)*	Activity
Nursing assistant	100	Supervision and assistance in daily life activities, promotion of autonomy in urinary function, feeding and mobility
Nurse	18	Specific competence needed in geriatric and dementia care. Detection of clinical changes. Regular contact with caregiver and GP. Treatment and prevention of geriatric syndromes
Physiotherapist	6	Functional assessment and evaluation of motor skills. Drawing up of individual rehabilitation plans. Promotion of group physical activity
Occupational therapist	18 (including occupational therapist and/or social health educator)	Psychosocial and rehabilitative interventions promoting cognitive stimulation, independence, reduction of BPSD and environmental adaptation. Focus on occupations meaningful for daily living and maintenance of autonomy
Social health educator		Psychosocial, educational and rehabilitative interventions promoting cognitive stimulation, psychological well-being, reduction of BPSD and environmental adaptation. Focus on social inclusion and integration into the community
Geriatrician or other dementia specialist	6	Pharmacological treatment of BPSD, prevention and treatment of geriatric syndromes, promotion of comprehensive assessment, staff training, contact with GP. Monitoring and encouraging vaccinations
Psychologist	6	Psychosocial interventions cognitive and/or emotion oriented, including cognitive stimulation therapy. Support of family and prevention of burnout of personnel
Qualified music therapist; qualified art therapist	6	Music therapy and art therapy projects (groups of 3–7 patients at least twice a week) aimed at decreasing depressive symptoms and BPSD and enhancing social skills
*Organizational aspects*		
Assistance and working times	The recommended number of day care recipients simultaneously present is 15–20, with a ratio of one operator for three patients. Additional hours should be forecasted for monthly meetings, discussion with caregivers, post-discharge return at home, integration of new care recipients	
Personnel selection	The selection should be performed according to specific attitudes and education, professional capacity and motivation. During opening hours, the presence should be ensured of at least one healthcare provider certified in BLS/AED in order to respond to an emergency and provide care	
Continuous staff training	The launch of the Italian Continuous Medical Education program in 2002 established the obligation of continuing education for Italian health professionals [43, 44] In addition to refresh courses, meetings with staff from other structures should be organized. Contact with local universities and tutoring of healthcare professional trainees should be encouraged [45]	

*BPSD* behavioural and psychological symptoms of dementia, *GP* general practitioner, *ICP* individualized care plan, *BLS* basic life support, *AED* automated external defibrillator

*Expected workload is intended for a standard Dementia Day Care Centre that accommodates 15 care recipients

The coordinator is responsible for interaction with healthcare district, GPs, local services and family members. The coordinator is also in charge of service organization, administration, monitoring of performance and of quality assurance. The care team as a whole is responsible for drafting and executing the ICP, including the identification of a case coordinator (case manager), who coordinates interventions included in the plan. Personnel included in the care with specific roles and expected workload for a standard (15 care recipients) DDCC are summarized in Table 2 [18, 41, 42].

## Psychosocial interventions

Scientific evidence is available supporting the efficacy of psychosocial interventions (PSIs) in reducing cognitive decline and BPSD and in promoting well-being and quality of life of people with dementia and their carers [46–48]. PSIs include activities aimed at enhancing preserved abilities and capacities of each patient, reducing social isolation and preserving self-esteem and personal identity [49].

PSIs can be grouped into four categories, according to their focus: cognition and function oriented, behaviour oriented, emotion oriented and sensory stimulation oriented (summarized in Tables 3a, b). Cognition- and function-oriented interventions (Table 3a) are aimed at maintaining cognitive and daily functioning as long as possible [46]. The group of behaviour-, emotion- and sensory stimulation-oriented interventions (Table 3b) has a crucial role for the treatment of BPSDs [50, 51] and should be considered as a first-line treatment in this setting [51], due to limited efficacy and increased risk of adverse events associated with antipsychotics. The evidence currently available on each intervention is summarized in the last column of the tables. All the listed interventions have been shown to have some positive effects, although, for some of them, the results are more uncertain than for others. Thus, all interventions could be delivered to dementia day care recipients, but the choice to apply one of them rather than another also depends on local factors (e.g. centre resources, space, available professionals) and on the preferences and specific requirements of the care recipients.

**Table 3:** Psychosocial interventions: main features and evidence of efficacy

*Psychosocial interventions: cognitive and function oriented activities*		
Intervention	Features	Evidence of efficacy
Cognitive training	Stimulation of specific cognitive areas through individual or group sessions	*Uncertain beneficial effect on memory in dementia *[46, 55]
Cognitive rehabilitation	Intervention aimed at cognitive functions necessary for daily living activities deemed relevant for the person	*In moderate dementia positive effects on stimulated cognitive function and on disability, delay of nursing home admission *[56]
Cognitive stimulation therapy	Structured stimulation of cognitive functions, emotions and social skills, oriented to patient's well being	*Improvement of cognitive abilities (comparable to cholinesterase inhibitors), social interaction and quality of life *[57, 58]
Physical activity	Specific programs for different stages of cognitive deterioration, possible more effective if combined with cognitive tasks	*Reduced decline of autonomy. Possible cognitive improvement associated with aerobic exercise. Non-consistent data on fall risk *[59–67]
Occupational therapy	Intervention based on meaningful activities of daily life	*Improvement of autonomy, quality of life, mood and caregiver’s stress. Short-term improvement of BPSD* [68–70]

*Psychosocial interventions: emotion, behaviour and sensory stimulation oriented activities*		
Intervention	Features	Evidence of efficacy
Systemic Intervention	Functional analysis of behaviour, including the identification of “unmet needs”. Person-centred-care	*Comparable effectiveness to drug therapies on decreasing agitation. Improvement of quality of life *[71–73]
Music-therapy Art therapy	Using music with a therapeutic purpose and for individually planned outcomes, including both active and receptive approaches [74–76] Using art as therapy, either referring to “art appreciation” programs or “making visual art” laboratories, aimed at enhancing well-being [77].	*Effectiveness of music therapy on reducing agitation, depression, anxiety, overall behavioural problems, and increasing quality of life *[78–81]; *limited or no evidence on cognitive function* [82] *Little evidence on effectiveness of dance-therapy* [83] *Art therapy improves neuropsychiatric symptoms, social behaviour and self-esteem in small trials* [77, 84, 85]
Animal-assisted therapy or activities carried out with the presence of people or objects	Using animals, familiar objects (dolls) or people (clown) for therapeutic purposes	*Benefits of animal-assisted therapy on anxiety, depression and agitation. Limited evidence on the psychological wellbeing of doll-therapy *[77, 86, 87]
Personally targeted activities	A wide range of activities and interventions that are important and meaningful to the person	*Possible efficacy on BPSD* [88, 89]
Multisensory stimulation	Use of multisensory environmental stimulation (snoezelen) or aroma. Therapeutic Garden (TG) providing sensory stimulation	*Possible efficacy on apathy, agitation, wandering and mood in severe dementia (snoezelen). Uncertain effects of aromatherapy on agitation * [90]. *TG may reduce BPSD* [30–32]

*BPSD * behavioural and psychological symptoms of dementia

The proposed activities should be simple and of limited duration, to avoid tiredness or stress; activities should also be person-centred according to stage of illness, needs and wishes of recipients. Therefore, a careful clinical, functional and cognitive assessment is required, coupled with the knowledge of personal history. The effectiveness over time of PSIs should be assessed through validated scales, such as the Neuropsychiatric Inventory [52] and the Cohen–Mansfield Agitation Inventory for behavioural assessment [53]. Moreover, the effects of PSIs on quality of life should be regularly evaluated through a scale validated for the specific level of dementia [54]. Examples of tailored, person-centred interventions are presented in a video recorded at the Monteoliveto Centre in Pistoia (https://www.youtube.com/watch?v=oGeP8nGCMRg).

## Drug treatment of BPSD

While PSIs must be considered as the first-line treatment of BPSD, the association of pharmacological interventions has a synergistic effect in improving neuropsychiatric symptoms, especially in long-term care [91]. Pharmacological intervention may be considered in case of severe BPSD unresponsive to non-pharmacological measures and when BPSD entail great suffering or risk [92].

Antipsychotics (APs) are still the therapy with more consistent documentation of efficacy in the short term, but with a risk of serious adverse events, including higher risk of mortality and stroke. Atypical APs are prescribed at least in 25% of patients with dementia [93]; since 2000 their use has been the object of warning about their safety in patients with dementia. In 2008, the warning was extended to typical APs too. The warning issued by the national and international regulatory authorities reports about an increased mortality rate in people with dementia treated with APs, with the risk being even higher with typical in comparison with atypical ones [94]. Therefore, when required, the use of atypical APs is still preferred over typical ones for the treatment of psychosis and aggressive behaviour in DDCCs [92]. Limited evidence is available regarding long-term use of APs. A prolonged treatment may be appropriate for cases with persistent and severe symptoms which show a good clinical response, although an attempt to reduce and eventually discontinue the therapy should be periodically considered [95].

Drug treatments of BPSD alternative to APs include selective serotonin reuptake inhibitors and trazodone, with citalopram having the strongest evidence for agitation, but with an increased risk of QT prolongation [96]. Other drugs with possible efficacy on BPSD include memantine [97], antiepileptics [98] and ginkgo biloba extract [99]. The use of analgesic drugs has been reported to reduce BPSD in nursing home residents [100].

According to available recommendations [92, 101, 102], psychopharmacological management in DDCCs should include the thorough appraisal of previous drug treatments, the assessment of type, frequency, severity and possible triggers of BPSD using standardized scales (e.g. Neuropsychiatric Inventory) and the possibility of drug de-prescription at admission and of a gradual dose tapering. Monotherapy should be preferred, starting treatment using the minimum effective dose, discontinuing after 2 weeks in the absence of an evident clinical response and attempting the interruption after 4 months in case of efficacy. A careful monitoring of possible adverse events should include the evaluation of new-onset or worsening of parkinsonism, immobilization and fall risk, presence of cardiovascular disease, QTc interval, serum potassium levels and co-occurring prescriptions. Exchange of information with the GP and the memory clinic on this issue is recommended.

## Prevention and treatment of geriatric syndromes

Geriatric syndromes are typical conditions of old age, resulting from physiological and pathological changes, with common risk factors, often coexisting in the same person and carrying the risk of worse prognosis [103].

The main geriatric syndromes that can be observed in DDCCs are listed below.

- *Falls* are frequent in people with dementia in long-term care and may lead to severe consequences, including fractures, head trauma, hospitalization, institutionalization, worsening disability and depression. Main risk factors for falls include sensory impairments, imbalance, specific drugs and polypharmacy, hypotension and environmental obstacles [104].
- *Delirium* is a dysfunction of awareness and attention, with fluctuating course during the day, that develops over a short period of time (hours to days), represents an acute change that cannot be explained by a coexisting neuropsychiatric condition, and is related to an underlying potentially reversible cause (acute medical condition drugs), although being often multifactorial. Delirium is frequent in dementia and should be recognized and differentiated from BPSD in DDCCs [105, 106].
- *Immobilization syndrome* is the consequence of interruption or reduction of physical activity [107], due to ageing-associated mobility disability and vulnerability of the organism. In dementia, even minor pathological conditions may limit physical activity.
- *Pressure sores* are ischaemic skin lesions caused by immobilization and worsened by malnutrition, urinary/faecal incontinence and peripheral hypoperfusion [108, 109].
- *Urinary incontinence* is highly prevalent in dementia and is associated with both psychological (reduction in self-esteem, depression and sleep disturbance), medical consequences (pressure sore risk), and increased care burden and risk of nursing home admission [110, 111].
- *Malnutrition* in dementia is typically represented by protein–calorie undernutrition due to low intake of nutrients, potentially leading to loss of muscle mass and strength (sarcopenia) [112] with consequent increased risk of falls and worsening disability, reduced wounds’ healing capacity, weakening of immune response and higher risk of mortality [113].
- *Dysphagia* is often associated with neuromotor signs (e.g. parkinsonism) and behavioural symptoms [114] and is typically present in severe stages of dementia. It increases the risk of aspiration pneumonia, dehydration, malnutrition and death [115].
- *Chronic and recurrent pain* is an unpleasant sensory and emotional experience and in people with dementia may be the cause of BPSD, such as agitation and aggression [116], and is also associated with the decline of cognitive function and functional status [117].

Table 4 itemizes the assessment tools and types of interventions (treatment and prevention) proposed for each geriatric syndrome.

**Table 4:** Assessment tools and treatment/prevention of geriatric syndromes

Syndrome	Assessment tools	Possible interventions
*Falls*	Collecting anamnestic information on previous falls, comorbidity and medications Assessment of orthostatic hypotension [118] Evaluation of lower limb performance (Tinetti Scale) and sarcopenia (SPPB) [112] Risk of fracture (FRAX)	Promotion of physical activity and rehabilitation (including use of mobility aids), revision of pharmacological treatment and therapies for osteoporosis. Removal of environmental risk factors and increased risk awareness of family members [118, 119]
*Delirium*	Confusion assessment method 4AT Test [120, 121]	Systematic detection of acute clinical events. Mobilization, correction of sensory deficits, cognitive and orientation stimulation, encourage hydration, psychopharmacological therapy change and pain management [122]
*Pressure sores*	Predicting pressure ulcer risk (Braden Scale) Detection of predisposing factors Staging of present lesions [109]	Regular mobilization, repositioning, use of pressure injury prevention devices, treatment of predisposing factors (urinary/faecal incontinence, undernutrition). Systematic skin inspection in patients at risk [109, 123]
*Urinary incontinence*	Collecting anamnestic information Bladder diary [124, 125]	Treatment of reversible forms. Prompted or scheduled voiding. Removal of environmental obstacles. Avoid the use of bladder catheter to manage urinary incontinence [126] Choice of appropriate absorbent pads [124, 125]
*Malnutrition*	Body weight and mid-upper arm circumference monitoring [127] Sarcopenia assessment using dynamometer [113] Mini Nutritional Assessment or Malnutrition Universal Screening Tool (MUST) [128, 129] Evaluation of eating behaviour (Eating Behaviour Scale) [130]	Adequate nutritional intake. Appropriate care strategies. Focus on dysphagia (see below). Consider protein and energy supplements [131, 132]
*Dysphagia*	Screening for dysphagia (water swallow test) [133] Detect clinical conditions possibly related with dysphagia, including drug use (e.g. antipsychotics) [115]	Adaptation of food texture and fluid consistency to severity of dysphagia. Focus on posture during meal, oral hygiene and dentures. Medication review. Implement staff knowledge about careful feeding by hand in severe stages [134]
*Chronic and recurrent pain*	History of pain or risk factors for chronic pain. Detection of behavioural pain indicators with scales validated in dementia (PAINAD) [135]	Treatment of causes when possible. Physical activity and physiotherapy. Pharmacological pain management [100]

*SPPB*  short physical performance battery, *FRAX* Fracture Risk Assessment Tool

## Vaccinations

Influenza and pneumococcal infections are recognized to be a significant health problem and cause of death, especially in fragile persons such as dementia people in DDCCs [136]. Vaccination is an effective measure to prevent the impact of these infections. Long-Term-Care Committee of the Society for Healthcare Epidemiology of America recommends in long-term care facilities (LTCF) residents should receive vaccination at the beginning of the influenza season unless a contraindication exists or the patient refuses [137, 138]. Moreover, the Society strongly supports influenza and pneumococcal vaccination of all healthcare personnel (HCP), including those who do not provide direct care [139]. Coronavirus disease (COVID-19) [140] and herpes zoster [141] vaccinations are also recommended.

## Infection prevention: the lesson from the COVID-19 pandemic

In addition to the devastating effects on people's lives, with hundreds of deaths per day in the first phase of the pandemic, since 2020 COVID-19 pandemic has deeply impacted on the care of older adults with Alzheimer’s disease. A recent review investigated the effects of the COVID-19 lockdown on neuropsychiatric symptoms, in people with dementia or mild cognitive impairment [142]. Data from 21 studies all around the world showed that symptoms such as depression, anxiety, agitation, irritability and apathy increased in this population during the lockdown, likely due to isolation and loss of services. The COVID-19 confinement also increased caregivers’ stress, independently of the dementia severity [143].

The serious effects of COVID-19 pandemic on both DDCCs users and services clearly emerged in a specific survey conducted in Italy, promoted by the Italian Group on Dementia Day Care Centres (unpublished data). Eighty-one out of 470 centres (private centres, *n* = 45; public centres, *n* = 36), mostly in Northern (*N* = 44) or Central (*N* = 30) Italy, filled the questionnaire. On the date of the survey (April 2021), 28 centres were still closed due to the pandemic, and 53 had resumed activity after a period of suspension. Among the active centres, only 10% retained the number of care recipients admitted as in the pre-pandemic period, whereas the number was reduced by more than 50%, or less than 50%, in 21 and 24 centres, respectively. Overall, there was a 60% reduction in the number of care recipients. Moreover, several centres had changed the number of days they were open and/or the opening hours.

As well as continuing to provide assistance in situations that require reduced physical contact, day care health workers must maintain their own safety and that of the users. To this purpose, guidelines have been drawn up by scientific societies and health authorities in individual countries [144] that will probably be modified according to the evolution of the pandemic. Specific procedures of containment are recommended, including:

(a) the periodic sanitization of environments;

(b) the identification of a “COVID-manager” for the prevention and control of healthcare-related infections; he/she must be a person, specifically trained for COVID-19, who knows the place of care and its problems according to the type of patients in the facility to observe, prevent and manage critical issues in real time;

(c) the monitoring of own body temperature by all healthcare workers before the start of the work shift, with a ban on entering the facility and starting the shift if a body temperature above 37.5 °C or other symptoms relevant to COVID-19 are detected;

(d) the obligation for caregivers to report the onset of symptoms (fever, cough, respiratory difficulties, anosmia, ageusia, etc.) of both the subject and the other cohabitants before accessing the day care centres;

(e) depending on the characteristics of the care recipients, the possibility of rotating attendance to encourage more people to take part in the activities by extending the opening hours; users can therefore also be organized in two daily shifts (morning and afternoon) according to the preferences expressed by caregivers; social distancing must be guaranteed in the same environment;

(f) the need to follow up remotely care recipients who cannot access the centre, to maintain contact and monitor clinical conditions;

(g) the rescheduling of many internal activities to ensure security; for example, occupational therapy, cognitive stimulation and physiotherapy activities should be carried out in small groups, in large, airy spaces or outdoors, trying to encourage the maintenance of social distance.

## Support and training for informal caregivers

Family and caregivers represent the main assistance resource for people with dementia. Over time, care needs gradually intensify, and therefore care modalities should undergo continuous and dynamic changes [145]. *Formal caregiver*s provide professional and paid assistance, while *informal caregiver*s, usually a family member (the spouse and/or children), give assistance voluntarily and for free. The latter experience an emotional and social, as well as physical, burden that can be alleviated by psychosocial and educational interventions [146, 147]. Unfortunately, though widely available in European countries, services of counselling, support and education for informal caregivers of people with dementia are rarely utilized [148].

Gradual personality changes in people with dementia, often resulting in alteration of the relationship with their caregivers, can represent the beginning of a process of atypical or anticipatory mourning for carers [149]. For these reasons, caregivers’ well-being should be cared for, with social support being an important protective factor [150] and specific interventions such as cognitive–behavioural therapy (CBT), including grief-specific strategies, should be taken into account [151].

Caregiver education is usually organized as a group activity, aimed at improving the adaptation of carers to assistance needs, establishing a healthy relationship, teaching coping strategies and promoting the interpretation of verbal and non-verbal language [152].

In DDCCs, it is possible to modify the level of caregivers’ stress [2], not only by the relief from daily assistance, but also with the treatment of BPSD and through support, information and training of family members. The working group within DDCCs should take care of family members since the admission to the service. A *caregiver assessment* should be coupled with patient assessment and includes schooling, work activity, degree of kinship and cohabitation with the patient, other relatives to assist, availability of other formal or informal caregivers, presence of interpersonal conflicts, and the level of physical, emotional and social burden. Care burden should be assessed at admission and reassessed periodically with specific scales, such as the Caregiver Burden Inventory [153]. The *interventions* offered by DDCCs to families include active listening, individuals or group interventions, education on the characteristics of the disease, including legal issues, support to relational approach at home, management of pharmacological therapy, and adaptation of home environment. During the COVID-19 pandemic, regular telephone contacts and videoconferencing have become common forms of care support, which have been appreciated by caregivers [154], and their use should be maintained by DDCCs, especially for subjects who attend the service only few days per week. Psychotherapy for caregivers may be indicated in selected cases. Moreover, DDCCs can be an appropriate setting to discuss advance care planning for severe stages of the disease [155].

## Conclusions

DDCCs should be considered an integral part of the treatment of dementia with behavioural and psychological symptoms, as they provide both psychosocial interventions for affected subjects and support for caregivers. The availability of an adequate physical environment, trained personnel and appropriate care approaches are crucial to attaining the therapeutic goals. The many existing centres in Italy are heterogeneous in terms of architectural features, organization, available professionals and interventions provided. Even most worrying are the inhomogeneous distribution and the overall shortage of such services. This paper is intended as a guide and a support for the organization of DDCCs and possibly for monitoring the quality of care delivered.
